# Furcal perforation repair using calcium enriched mixture cement

**DOI:** 10.4103/0972-0707.71650

**Published:** 2010

**Authors:** Saeed Asgary

**Affiliations:** Iranian Center for Endodontic Research (ICER), Iran Center for Dental Research (ICDR), Dental School, Shahid Beheshti University of Medical Sciences, Tehran, Iran

**Keywords:** Biocompatibility, calcium enriched mixture, CEM cement, NEC, perforation repair

## Abstract

This case describes a furcal perforation in a mandibular first molar accompanied by furcal lesion which has been managed after one month. Root canal treatment was performed; subsequently, the defect was repaired with calcium enriched mixture (CEM) cement. The endodontically treated tooth was restored with amalgam. A 24-month recall showed no evidence of periodontal breakdown, no symptoms, in addition to completes healing of furcal lesion. According to physical and biological properties of the newly introduced CEM cement, this novel material may be suitable for sealing and repairing the perforations. The present case report supports this hypothesis.

## INTRODUCTION

Furcal perforations are major iatrogenic complications and could lead to endodontic failure.[1,2] Prognostic indicators that influence the treatment outcome are time of repair, size, level, and location of the perforation, presence of periodontal disease and pre-endodontic pulp vitality status.[1,3]

On the basis of the particular characteristics of furcal perforations, they can be managed either surgically or non-surgically.[4] The prognosis is generally excellent if the problem is well diagnosed and well-performed repair with a material which can provide proper sealing ability and biocompatibility.[5]

Amalgam, super EBA, Cavit, glass ionomer, light cure composite resin, calcium hydroxide, and mineral trioxide aggregate (MTA) have been used with different degrees of success for perforation repair.[4,6–8] Several studies done on MTA, as a relatively new material, have shown excellent biocompatibility when used to repair furcal perforations.[9] However, long setting time, difficulty in handling, and relatively high price are some disadvantages of this material.[10,11]

Recently, calcium enriched mixture (CEM) cement has been recommended as an appropriate root-end filling material.[12] CEM cement demonstrated good treatment outcomes in direct pulpal capping.[13] Histological observations after repair of furcal perforation with CEM cement in dogs have shown not only a re-establishment of normal periodontium, but also cementogenesis over the material.[14] This material has also shown favorable results in apexogenesis[15] as well as pulpotomy of permanent human molars with established irreversible pulpitis and management of internal root resorption.[16]

CEM cement has antibacterial effects better than MTA and comparable with calcium hydroxide;[17] it has also low cytotoxic effect similar to MTA.[18] Results of recent studies indicate that mixed CEM cement releases calcium and phosphate ions[19] and then forms hydroxyapatite not only in simulated body tissue fluid but also in normal saline solution; the latter of which is unlike MTA.[20] This novel cement has similar pH, increased flow, but decreased working time, film thickness, and lower estimated price than MTA.[11]

The following case verifies that CEM cement is an appropriate material for furcal perforation repair in patients.

## CASE REPORT

**Figure 1:**
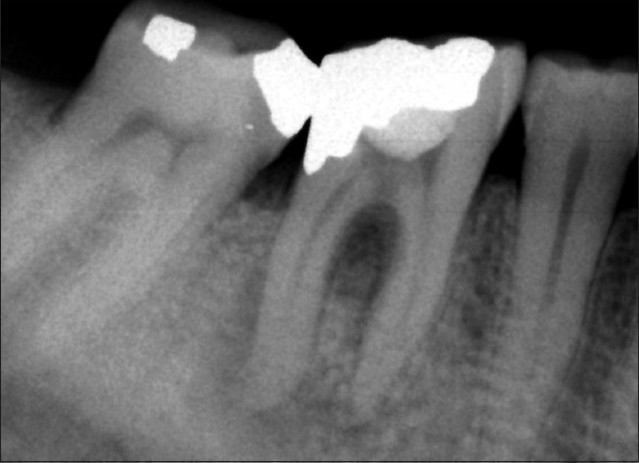
Initial periapical image of left first mandibular molar showing furcal perforation and apical lesions

A 39-year-old male referred to the private endodontic clinic with an acute abscess adjacent to the left first mandibular molar. Clinical examination showed that the tooth was tender to percussion, sealed coronally by temporary cement, and exhibited slight mobility. Radiographs showed a furcal perforation, resulted in a lesion. Two apical radiolucencies were also observed, which implied pulp necrosis [Figure 1]. The medical history was non-contributory. Treatment options which were indicated for the tooth were extraction and surgical/non-surgical repair of the perforation. Regarding the patient preference in saving the tooth via a non-surgical procedure, furcal perforation repair with CEM cement was chosen.

**Figure 2:**
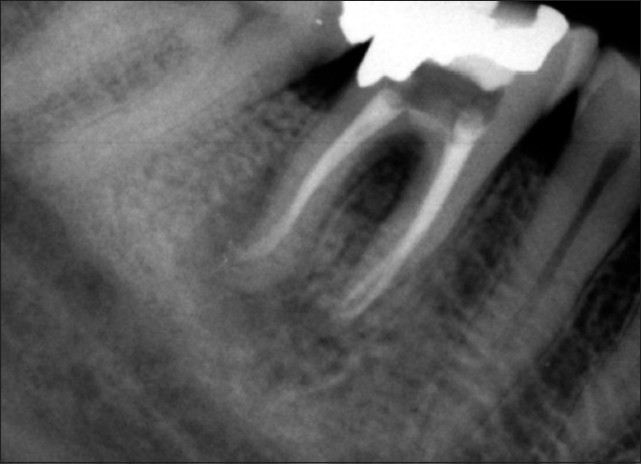
Periapical image after treatment

**Figure 3:**
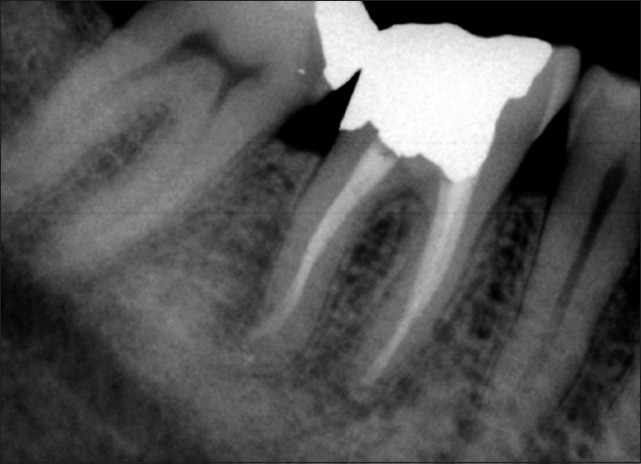
At 2-year follow-up, there is complete osseous healing at the apex and the bifurcation

After administration of local anaesthesia (Lidocaine 2% with epinephrine 1:80000; Daroupakhsh, Tehran, Iran) and with a rubber dam in place, the temporary restorative material was removed and a correction in access cavity was made. Cotton pellet was placed in the orifice of perforation. The canals was cleaned and shaped using Profile 0.04 files (Dentsply, Maillefer, OK, USA) by step-back flaring technique and constant irrigation with 2.5% sodium hypochlorite and then obturated with gutta-percha points and Roth 801 root canal sealer (Roth International, USA) using lateral condensation. The perforation site was irrigated with 2.5% sodium hypochlorite and normal saline. CEM cement (BioniqueDent, Tehran, Iran) was prepared according to inventor’s instruction and was placed into the pulp chamber with an amalgam carrier, and gently packed with a cotton pellet to obtain a good adaptability [Figure 2]. The cement was covered with a moistened cotton pellet and Cavit temporary restoration material (ESPE America, INC., Norristown, PA, USA). At the 1 and 7-day follow-ups, the patient was asymptomatic and his pain and swelling subsided. He was referred for coronal restoration of endodontically treated tooth. Two years after treatment, radiography showed a complete osseous healing at the apices and the furcation region as well [Figure 3].

## DISCUSSION

The time lapse between perforation and repair is one of the most prognostic factors for success of perforation repairs.[21] The patient in the present case report was referred to endodontic clinic one month after the furcal perforation. He presented a bifurcal perforation in the left first mandibular molar, probably because of an erroneous access preparation. Such perforations lead to injury of periodontium in the bifurcation, bacterial contamination, formation of inflammation and bone resorption.[22,23] Therefore, treatment outcome of the furcal perforation depends on prevention of microleakage and control of tissue inflammation *via* sealing of the perforation site with a biocompatible material. The success of the treatment with CEM cement is attributed to removal of contaminants as well as cleaning of the pulp chamber, perforation, and wound site with copious amounts of 2.5% NaOCl before placement of this cement. Besides, it is likely that the antibacterial effect[17] in addition to high pH[11] of CEM cement contributed to further bacterial reduction.

The treatment of the presented case was performed single-visit; however, two-visit RCTs have been previously popular for treatment of non-vital tooth with endodontic lesions. The documented evidence shows that the success rate of one-visit RCT in such teeth is similar or even better than multiple-visit RCT.[23,24] Intracanal medicament which has been previously suggested for management of intracanal infections was not used in this case; this is because recent documents show that using calcium hydroxide (as the gold standard) for interappointment dressing does not necessarily provide higher success rate.[25,26]

There is a general agreement on the fact that material’s ability to seal the cavity from further bacterial ingress is the key to success.[9] Short-term healing of acute abscess and complete resolution of furcal lesion at two-year follow up in the present case indicate absence of leakage at the site of the perforation. It has been reported that the sealing ability of the CEM cement was comparable to MTA as root-end filling material.[12]

One of the interesting radiographic features was formation of normal PDL [Figure 3], indicating the biocompatibility of the CEM cement. This feature can, in turn, be attributed to low cytotoxic effect of CEM cement on different cell lines,[18,27] which promotes osteogenesis and cementogenesis,[14] which allow regeneration of the PDL around the site of injury. Additionally, this novel cement released calcium and phosphorus ions from indigenous sources result in a rich pool of OH^−^, Ca^2+^ and PO4^−^ ions.[19,20] These elements are used in the process of hydroxyapatite (HA) production. Furthermore, a recent SEM study showed that distribution pattern of calcium, phosphorus, and oxygen in the surface of the CEM cement was comparable to that of surrounding dentin.[28] This finding indicates that the composition of the cement is similar to dentin. HA is a main component of dentin; therefore, similarity between CEM cement and dentin might help the cementogenesis over it.[14] This feature is hypothetically responsible for its biocompatibility and its other optimum specifications.

CEM cement was considered as the material of choice based on the results of *in vivo* studies which revealed that CEM cement is able to stimulate dentinogenesis after direct pulp capping[13] and pulpotomy in animals[29] and humans,[16] apexogenesis[15] and also cementogenesis after perforation repair or surgery.[14,30] If the positive result of this “delayed repair of furcal perforation” in human beings is this good, there would seem to be promise for use of the CEM cement in a timelier manner with recent perforations.

After two years, the tooth remained asymptomatic with no clinical signs of pathology; radiographic examination showed signs of normality and complete osseous repair, and the patient was satisfied of saving the tooth. More cases are needed to substantiate the effectiveness of CEM cement for repair of furcal perforations, but early indications are promising enough to suggest its use.
